# A novel metabarcoding diagnostic tool to explore protozoan haemoparasite diversity in mammals: a proof-of-concept study using canines from the tropics

**DOI:** 10.1038/s41598-019-49118-9

**Published:** 2019-09-02

**Authors:** Lucas G. Huggins, Anson V. Koehler, Dinh Ng-Nguyen, Stephen Wilcox, Bettina Schunack, Tawin Inpankaew, Rebecca J. Traub

**Affiliations:** 1 Faculty of Veterinary and Agricultural Sciences, University of Melbourne, Parkville, VIC 3052 Australia; 2 Faculty of Animal Sciences and Veterinary Medicine, Tay Nguyen University, Buon Ma Thuot, Dak Lak 630000 Vietnam; 3 Walter and Eliza Hall Institute of Medical Research, Parkville, VIC 3052 Australia; 4 Bayer Animal Health GmbH, Leverkusen, Germany; 5 Faculty of Veterinary Medicine, Kasetsart University, Bangkok, 10900 Thailand

**Keywords:** Microbiome, Microbial ecology, Microbiology techniques, Metagenomics

## Abstract

Haemoparasites are responsible for some of the most prevalent and debilitating canine illnesses across the globe, whilst also posing a significant zoonotic risk to humankind. Nowhere are the effects of such parasites more pronounced than in developing countries in the tropics where the abundance and diversity of ectoparasites that transmit these pathogens reaches its zenith. Here we describe the use of a novel next-generation sequencing (NGS) metabarcoding based approach to screen for a range of blood-borne apicomplexan and kinetoplastid parasites from populations of temple dogs in Bangkok, Thailand. Our methodology elucidated high rates of *Hepatozoon canis* and *Babesia vogeli* infection, whilst also being able to characterise co-infections. In addition, our approach was confirmed to be more sensitive than conventional endpoint PCR diagnostic methods. Two kinetoplastid infections were also detected, including one by *Trypanosoma evansi*, a pathogen that is rarely screened for in dogs and another by *Parabodo caudatus*, a poorly documented organism that has been previously reported inhabiting the urinary tract of a dog with haematuria. Such results demonstrate the power of NGS methodologies to unearth rare and unusual pathogens, especially in regions of the world where limited information on canine vector-borne haemoparasites exist.

## Introduction

Protozoan haemoparasites generate some of the highest rates of morbidity and mortality in canines worldwide, whilst some are also zoonotic, capable of producing significant infections in humans as well^1–4^. The principal taxonomic groups responsible are the bloodborne piroplasmids and kinetoplastids which are transmitted by haematophagous arthropods, such as ticks, fleas, sand-flies and mosquitoes, as vector-borne diseases (VBDs)^3,5^. Examples of haemoparasite zoonoses include leishmaniasis which has long been identified as an important canine VBD with a widespread, and in some regions expanding distribution^6,7^, whilst non-zoonotic diseases such as canine, equine or bovine babesiosis are nevertheless critically important diseases from a veterinary standpoint, with some species now recognised as key emerging pathogens^8,9^.

Apicomplexan *Babesia* spp. parasites are transmitted by tick vectors which invade erythrocytes and cause a spectrum of anaemia-related pathology depending on the species, from the relatively benign *Babesia vogeli* to the more virulent *Babesia canis* and *Babesia rossi* species^1,10^. Whilst it has not been confirmed that canine-infecting *Babesia* spp. can infect people, other members of the genus, including *Babesia microti* present a severe zoonotic threat^8,11^. In the tropics, kinetoplastid parasites such as *Trypanosoma evansi*, that are important livestock pathogens, can also frequently produce fatal infections in dogs^4^. Furthermore, canines are the primary zoonotic reservoir for *Leishmania infantum*, a kinetoplastid capable of causing a visceral, multi-organ disease in dogs and immunocompromised humans and children, particularly in regions of South America, the Middle East and the Mediterranean^12–14^.

Many of these haemoparasites are united by their ability to create enduring infections, that can last years, with periods of immunological control followed by remission^10,15,16^. This tenet facilitates the formation of a haemoparasite microbiome as a single host accumulates more infections, including those from bacteria, viruses and metazoans, some of which can be chronic and others short lived. Within the context of the canine blood microbiome a single haemoparasite may not be lethal, but still exert a toll on the dog in which it resides that may make the host more susceptible to other VBDs or make the pathogenesis of another parasite worse^17,18^. For instance, *Hepatozoon canis*, typically generates a subclinical infection^15^, however, when found to be coinfecting with *Babesia* spp. or bacterial VBDs a much more severe anaemia and overall disease outcome is generated^18^. Taking this into consideration, canine VBD diagnostic methods must be able to characterise the entire haemoparasite microbiome and not just a dominant pathogen within a host.

With the advent of next-generation sequencing (NGS) technologies the field of parasite diagnostic tests is in the process of being transformed. Conventional PCR (cPCR) methodologies such as endpoint or quantitative PCR, which themselves superseded laborious microscopy and culture-based methods, have always been limited in their assessment of microbiomes by the need for *a priori* data on a target species taxonomic barcoding sequence^19,20^. This restrains such methods to only detecting known and genetically characterised species, whilst ignoring rare or undiscovered species^19^. Additionally, endpoint PCR coupled with Sanger sequencing is typically unable to detect more than the dominant sequence in an amplicon of potentially many, thereby making the technique of limited utility for recognising mixed infections^21^. NGS-based diagnosis mitigates such limitations as class, phylum or even kingdom specific primers can be used to amplify around barcode regions that are unique to each species. Amplified DNA from all the different species barcodes in a sample can be sequenced in massive parallelisation, generating a sample metabarcode of every species present from a taxonomic group of interest, thereby elucidating an entire microbiome from within a specific host environment^22,23^.

The aim of this study was to develop an NGS-based diagnostic tool to fully characterise the apicomplexan and kinetoplastid haemoparasite microbiome from blood DNA samples, thereby including the ability to detect novel, rare or poorly documented species. Moreover, we aimed to compare this novel NGS-based method to the sensitivity and detection range of conventional PCR methods. Semi-domesticated and temple community canine populations from Thailand were chosen as there is relatively limited information regarding haemoparasite infection in Southeast Asian dogs, whilst the few studies that have been conducted have found parasite prevalence to be high^24–26^.

## Results

### Design of metabarcoding primers for Apicomplexa and Kinetoplastida

Two primer pairs were designed to exclusively amplify 18S rRNA sequences from the phylum Apicomplexa and the class Kinetoplastida using a diverse range of sequences from GenBank. As the metabarcoding was to be carried out on canine blood samples, Apicomplexa and Kinetoplastida sequences from common blood-infecting species were chosen (for the complete list see the “Methods” section). Primers were designed to bind to highly conserved 18S rRNA sequences but flanking areas of high sequence diversity (barcode regions) to provide species-level discrimination. Host, canine 18S rRNA sequences were also included in the alignment to ensure that the designed primers did not cross-react with canine DNA.

### Metabarcoding assay validation

Confirmed haemoparasite positive controls were used to find optimal PCR conditions, whilst primer cross-reactivity was tested for, using positive controls from species outside of the taxa targeted by each primer pair (see the “Methods” section). Mock communities were also generated by mixing haemoparasite positive controls. After completion of our developed metabarcoding pipeline these mock communities were consistently and accurately reflected in the final results.

### Bioinformatic analysis of Apicomplexa data

In total 6,649,169 (median 62,120) raw paired-end reads were obtained for the 104 multiplexed Apicomplexa amplicons, including two positive and two negative controls. After the DADA2 quality filtering, dereplication, chimera removal and pair-joining step, a total of 564,332 joined sequences were retained, representing a retention of 16.97% of original reads (564,332 × 2 ÷ 6,649,169).

### NGS characterisation of Apicomplexa

**Figure 1:**
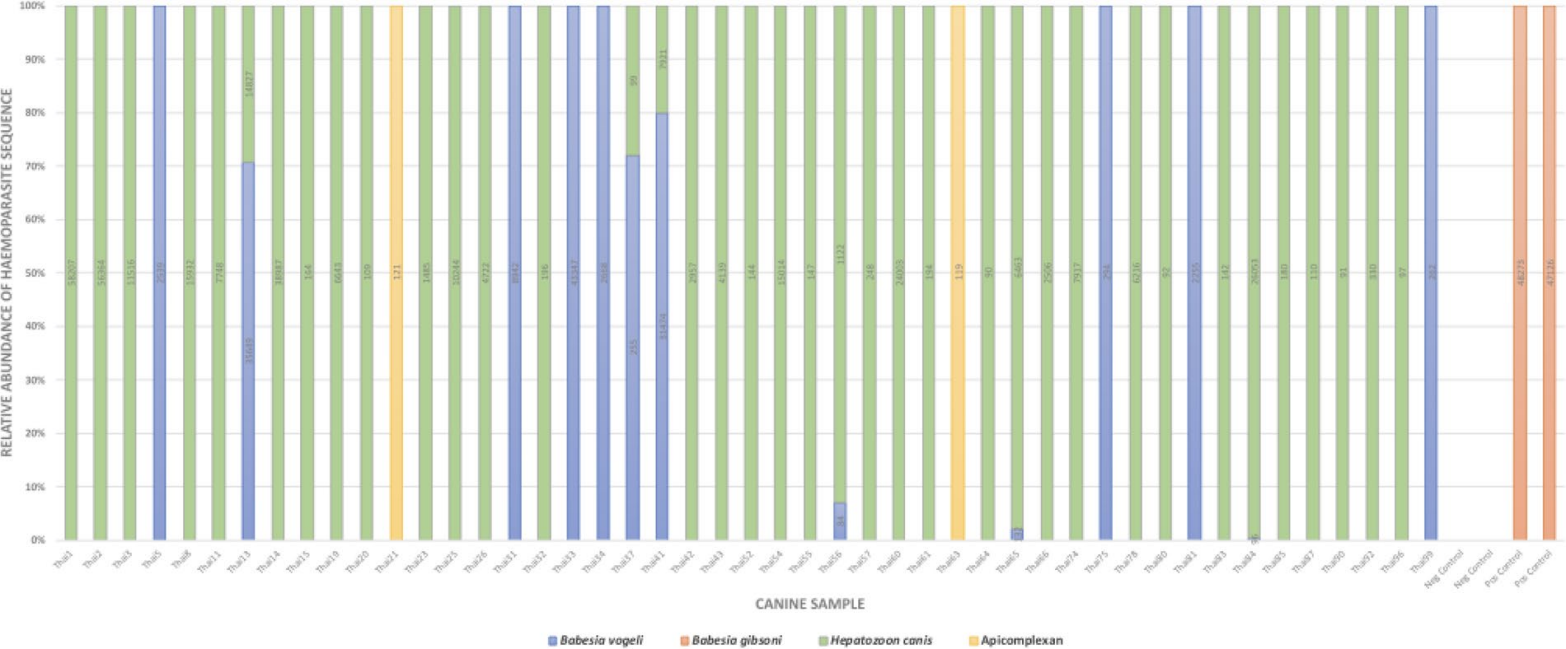
Relative sequence composition of canine blood samples found to be positive for an apicomplexan infection. Numbers in columns represent actual read counts.

Of the 100 canine blood samples tested 13 were found to be positive for *B*. *vogeli* (mean reads 9,846; range 84–43,347) and 38 for *H*. *canis* (mean reads 8,774; range 90–58,207); six of these dogs were infected with both haemoparasite species. Two canine DNA samples returned sequences that could only be identified to the level of phylum Apicomplexa (mean reads 120; range 119–121). When this sequence was run through a BLASTn search it returned a 100% identity result with a diverse range of sarcocystidae family pathogens, making species level assignment impossible with this sequence alone. In total 47% of dogs were found infected with at least one apicomplexan VBD via deep sequencing (Fig. 1).

Classification of NGS Amplicon Sequence Variants (ASVs) by the scikit-learn classifier was supported by phylogenetic analysis (Supplementary File 1; Figs S1 and S2) which demonstrated ASVs classified as *H*. *canis*, and *B*. *vogeli* clustered with high posterior probability between the relevant reference sequences taken from GenBank.

ASVs classified to species had higher nucleotide homology to reference sequences and less sequence variation than sequences assigned to higher taxonomic levels. For example, across all apicomplexan species used in our primer design alignment the mean nucleotide pairwise identities in the 130 bp region amplified by our primers was 71% ± 16.4% (mean ± standard deviation). In comparison, the 10 ASVs classified as *H*. *canis* had a mean nucleotide pairwise identity of 98.8% ± 0.4% when compared to six *H*. *canis* 18S rRNA reference strains (GenBank accession numbers in Supplementary File 1; Fig S1). The four ASVs classified as *B*. *vogeli* had a mean nucleotide pairwise identity of 99.0 ± 0.5% when compared to four *B*. *vogeli* reference strains (GenBank accession numbers in Supplementary File 1: Fig S2). This demonstrates the range of nucleotide diversity within our apicomplexan barcode region, and the observed inter- and intraspecies nucleotide diversity that was used to accurately taxonomically classify each ASV.

Infections were considered true by NGS if a sample had a VBD read count of over 44. This threshold was calculated as the mean reads of two canine DNA samples that were identified as having sequences from the positive controls used, potentially due to index misreading or hybridisation errors during Illumina sequencing^27^. This was supported by observation of where on the 96-well plate the samples with positive control sequences appeared, which showed substantial distance from the positive control locations. The mean Phred quality score over the adapter and indexing regions for the raw data was 31, highlighting a base call error rate of between one in 1,000 to 10,000, that may have potentially led to occasional index misreading. This conclusion was further corroborated by separate cPCR reactions to target canine piroplasms^28^ that found the two samples that were thought to contain positive control sequences, as negative for piroplasm DNA.

### Comparison of NGS diagnostic methods with conventional PCR for Apicomplexa

A highly specific canine piroplasm nested PCR^28^ capable of amplifying all canine *Babesia* species found 13 blood samples to be infected with this pathogen, whilst a *H*. *canis* specific cPCR^29^ found 17 samples to be infected with this VBD. The results of both cPCR results taken together identified four dogs as being infected with both *Babesia* spp. and *H*. *canis*.

Table 1 shows the relevant agreement statistics between the two diagnostic tests. The Kappa statistic for the piroplasm specific cPCR showed very good agreement with the NGS results, whilst the Kappa value when comparing the *H*. *canis* PCR was fair. The NGS method demonstrated superior sensitivity regarding its ability to detect *H*. *canis* infections, identifying 21 more positives than the cPCR method.

**Table 1:** Apicomplexa NGS and conventional PCR (cPCR) agreement statistics.

VBD	cPCR	Apicomplexa NGS		Total Agreement (%)	Kappa* (agreement statistic)	Kappa SE
		POS	NEG			
*Babesia* spp.	**POS**	12	1	98	0.912 (Very good)	0.062
	**NEG**	1	86			
*Hepatozoon canis*	**POS**	15	2	75	0.406 (Fair)	0.088
	**NEG**	23	60			

*Kappa agreement level: K < 0.2 Poor; 0.21–0.40 Fair; 0.41–0.60 Moderate; 0.61–0.80 Good; 0.81–1.00 Very good.

### Apicomplexa NGS cross-validation

To cross-validate the taxonomic assignment provided by scikit-learn within the bioinformatics pipeline, endpoint PCR experiments were conducted on larger taxonomic barcode regions followed by Sanger sequencing. Amplicons produced by the piroplasmid specific PCR^28^ achieved a 100% query cover and identity match with *B*. *vogeli* isolate 68SR (GenBank accession no. MH100721.1) using the GenBank BLASTn tool. *H*. *canis* taxonomic classification was confirmed by a nested PCR designed in the present study, using Sarc-int-2F and Sarc-int-2R primers (Table 2), returning a 100% query cover and 99.26% identity match with *H*. *canis* voucher Junagadh 590 (GenBank accession no. MH922768.1).

**Table 2:** Apicomplexa and Kinetoplastida primers used for cross-validation of NGS results.

Apicomplexa Taxon Targeted	Primer Pair	Gene Targeted	Product Size	Reference
Canine piroplasm nested PCR	BTF1 & BTR1 BTF2 & BTR2	18S rRNA gene	930 bp 800 bp	^28^
*Hepatozoon canis*	HepF & HepR	18S rRNA gene	666 bp	^29^
Coccidia	COC-1 & COC-2	18S rRNA gene	280–350 bp	^30^
*Toxoplasma gondii*	Unnamed (see reference)	*T*. *gondii* 529 bp repeat element	529 bp	^32^
Tissue coccidia	COX10F & COX500R	Cytochrome c oxidase I (COX1)	470–510 bp	^31^
Coccidia specific nested PCR	Sarc-int-2F* (5′-AGCTCGTAGTTGGATATCTGCTG-3′) & Sarc-int-2R* (5′-CCTATCTTGTTATTCCATGCTGCA-3′)	Cytochrome c oxidase I (COX1) Uses PCR product from COX10F & COX500R primers	150 bp	This study*
**Kinetoplastida Taxon Targeted**	**Primer Pair**	**Gene Targeted**	**Product Size**	**Reference**
Kinetoplastida specific	Kin24SF & Kin24SR	24S alpha-subunit rRNA	440–520 bp	^37^
*Trypanosoma evansi*	RoTat1.2 F & RoTat1.2 R	*T*. *evansi* 1.2 Variable Surface Glycoprotein (VSG)	205 bp	^38^
Kinetoplastida specific	KinSSUF1 & KinSSUseqR2	18S rRNA	650 bp	^36^
Kinetoplastida specific nested PCR	PCaud1F* (5′-CTACCACTTCTACGGAGGGC-3′) & PCaud1R* (5′-GCACCAGACTTGTCCTCCAA-3′)	18S rRNA. Uses PCR product from KinSSUF1 & KinSSUseqR2 primers	130 bp	This study*

Asterisks denote nested PCR primers designed in the present study, thermocycling reagents and conditions for these primers are as detailed in the Kinetoplastida metabarcoding methods section with a lower annealing temperature of 52 °C.

For the two canine samples that had sequences that could not be classified below the taxonomic level of Apicomplexa, two different tissue coccidia-specific PCRs were conducted^30,31^. These were chosen as the apicomplexan sequence provided by NGS was found to be highly conserved across the sarcocystidae family by a BLASTn search. Unfortunately, no amplification could be achieved with these endpoint PCRs nor with a *T*. *gondii* specific real time PCR^32^, which was a suspected pathogen.

### Bioinformatic analysis of Kinetoplastida data

In total 4,457,913 (median 43,188) raw paired-end reads were obtained for the 104 multiplexed Kinetoplastida amplicons, including two positive and two negative controls. After the DADA2 quality filtering, dereplication, chimera removal and pair-joining step a total of 117,262 joined sequences were retained, representing a retention of 5.26% of original reads (117,262 × 2 ÷ 4,457,913).

### NGS characterisation of Kinetoplastida

Out of the 100 canine blood samples only two demonstrated amplification of sequences from potential haemoparasites. One sample had reads taxonomically assigned to *T*. *evansi* (18 reads), whilst the other was identified as *Parabodo caudatus* (126 reads). Both *Trypanosoma theileri* clade positive controls were successfully amplified and detected by the NGS diagnostic test.

Classification of NGS ASVs by the scikit-learn classifier was supported by phylogenetic analysis which demonstrated that the ASV classified as *T*. *evansi* clustered with high posterior probability between *T*. *evansi*, *Trypanosoma equiperdum* and *Trypanosoma brucei* reference sequences (Supplementary File 1; Fig. S3), the latter two are known to be indistinguishable at the 18S rRNA gene^33,34^. Nonetheless, *T*. *evansi* is the most likely infective agent in this case, as *T*. *equiperdum* is a horse-specific venereal pathogen, whilst *T*. *brucei* is geographically only found in Africa^33,34^. The ASV classified as *P*. *caudatus* clustered with *P*. *caudatus* and *Bodo caudatus* reference sequences (Supplementary File 1; Fig. S4), *Bodo* being the former genus name for this species^35^.

### Kinetoplastida NGS cross-validation

Two Kinetoplastida specific PCRs^36,37^ and one *T*. *evansi* specific PCR^38^ all failed to amplify the sample that was found to be *T*. *evansi* positive by NGS, without any product generated for sequencing. However, a nested PCR designed in the present study using PCaud1F and PCaud1R primers (Table 2) amplified a region from which 41 nucleotides achieved a 95% identity hit with two *Trypanosoma* spp. entries (GenBank accession numbers: JN315385.1 and AF359482.1). The same endpoint PCRs all failed to amplify from the sample found to be *P*. *caudatus* positive by NGS^36–38^. Blood smears from individuals identified as having Kinetoplastida DNA were also assessed microscopically for the presence of relevant organisms but returned negative results.

## Discussion

Our novel NGS-based diagnostic tool was able to thoroughly characterise the apicomplexan and kinetoplastid haemoparasite microbiome from canines and was demonstrated to be more diagnostically sensitive than conventional PCR on field samples. The developed methodology is not necessarily limited to canine application alone and may show utility in characterising protist pathogens from the blood of a range of animals, including humans. Our diagnostic method’s ability to detect unusual haemoparasites from blood, further highlights the power of this methodology to uncover new species or emerging disease threats of veterinary and clinical importance from countries which have so far had limited relevant research done within their borders.

Not only was our NGS method demonstrated to have a large breadth of taxonomic detection ability but was also proven to be highly sensitive. Concordance between the NGS method and *Babesia* specific cPCR was almost 100% with a Kappa statistic that indicated a very good level of agreement between the two methodologies. However, when our method was compared to the *Hepatozoon* specific PCR^29^ it greatly outperformed it, finding 21 more single *H*. *canis* infections with a Kappa statistic demonstrating a fair level of agreement between these tests. This superior sensitivity using an NGS-based method compared to cPCR has been demonstrated previously, in particular when targeting the bacterial pathogen *Anaplasma platys* from canine blood^39^.

*Babesia* species are important canine pathogen capable of producing grave morbidity or mortality in their hosts, particularly in puppies^4,10,11^. The 13% of dogs found to be infected in the present study largely supports that of other haemoparasite molecular surveys for the Southeast Asia region. *Babesia* infection rates range from 7.14% of stray dogs in The Philippines^40^, to 9.4% in Thailand^25^ and as many as 32.7% of semi-domesticated dogs in Cambodia^24^. In comparison, *H*. *canis* is a less virulent haemoparasite but was found in greater abundance than *B*. *vogeli* in the present study^15^. The 38% of dogs infected with *H*. *canis* by our NGS diagnostic tool was higher than other studies done in the country and SE Asia region. For example, a *H*. *canis* infection rate of 18.8% was found in Thailand^25^, whilst 10.9% of community dogs in Cambodia had been found to have this pathogen previously^24^. In addition, the *B*. *vogeli* and *H*. *canis* co-infection rate of 3.6% that was found in Cambodia^24^ also closely matches the 6% elucidated by NGS in the present study. Future work could benefit from the collection of data on haemoparasite vectors, such as ticks, to assess for potential correlations between local levels of haemoparasite infection and vector abundance.

Our NGS method detected two more *H*. *canis* and *B*. *vogeli* mixed infections than cPCR screening. Thorough detection of the complete haemoparasite microbiome is particularly pertinent given that there is a substantial body of evidence demonstrating more severe pathology and increased lethality is brought about by multiple VBD infections in a single host^18,41–44^. The better sensitivity of our NGS method at detecting such co-infections means that particularly at-risk individuals are not missed and can be prioritised. The relative sequence composition of these co-infections can be observed in Fig. 1. This bar graph displays three co-infections as being dominated by over 90% *H*. *canis* reads, whilst the others comprise over 70% *B*. *vogeli* reads. Such data may be indicative of the comparative parasitaemia of each VBD at the time of sampling.

Whilst there were only two kinetoplastid infections across the entire sample set, the detection of these was significant as *T*. *evansi* is a frequently lethal pathogen of dogs^4,45^ and *P*. *caudatus* has been seldom documented from canines with an unknown role as a potential infectious agent^36^. *T*. *evansi* is the aetiological agent of the damaging livestock disease ‘Surra’ which afflicts horses, cattle and camels but can also infect dogs, and various wildlife species via transmission using tabanid fly vectors^45^. *T*. *evansi* has only been identified from a dog in Thailand once before^46,47^ and given its severe pathogenicity is thus an important finding, demonstrating potential spill-over transmission from local livestock into canine hosts.

*P*. *caudatus* has been previously implicated in a case of gross haematuria in a canine, with these biflagellated protozoans being observed under the microscope from urine samples voided by an infected dog^36^. Similar pathogens have also been documented in human urine samples^48^, whilst closely related *Bodo* species have been found in the blood of the marsupial, *Bettongia penicillate*^49^ and in bats^50^. Whether or not the presence of such kinetoplastid DNA demonstrates them to be true pathogens or simply commensal organisms is debatable. In the case of the present study, the detection of *P*. *caudatus* within the blood microbiome is particularly notable as it is hard to rationalise how this typically environmental organism^51^ could have entered and then persisted in the canine bloodstream. Nonetheless, it is possible that the presence of *P*. *caudatus* sequences could be the result of environmental contamination, although this is unlikely given the thorough sterilisation and aseptic protocol utilised when collecting blood samples. If we have identified a *P*. *caudatus* bloodstream infection, the battery of different conventional PCRs that would have needed to be conducted to detect these unusual protozoa using traditional molecular methods would have been great, demonstrating the advantages of NGS-based analysis to find atypical pathogen species.

For the detection of *T*. *evansi* our 44 read cut-off was not applicable as these reads were unique to one sample and thus could not represent indexing cross-talk errors, our 18 *T*. *evansi* reads were also higher than a whole dataset threshold of 10 reads used in similar studies exploring eukaryote diversity via metabarcoding^52^. Our *T*. *evansi* diagnosis was later partially supported by a separate nested PCR developed in the current study with Sanger sequencing that returned a 41 bp run with 95% identity to *Trypanosoma* spp. However, the small size and only genus level taxonomic assignment of this corroboratory cPCR experiment, provides only limited cross-validation for our NGS method’s result of a *T*. *evansi* infection in this individual.

Endpoint PCR cross-validation for *P*. *caudatus* could not be achieved at all, possibly due to a very low concentration of circulating *P*. *caudatus* DNA or a dearth of publicly available 18S rRNA sequences for this species, making design of primers to target this organism suboptimal. Future experiments could reconduct NGS at a greater sequencing depth on samples that achieved low read counts for parasite DNA, to assess if results remain consistent and provide further support for the sensitivity of the existing metabarcoding protocol.

No evidence of the important zoonotic pathogen *Leishmania* was found in the canines tested in the present study, despite cases of human leishmaniasis being reported in Thailand caused by both *Leishmania martiniquensis* and *Leishmania siamensis*^53^. As yet, there is little-to-no evidence of canines acting as a reservoir of human-infecting *Leishmania* species in SE Asia, although in one study it was reported that local medical practitioners indicated a potential for dogs and rats to be acting as a source of human infection^54^.

The accuracy of species level taxonomic classification by the QIIME2 sci-kit learn classifier was supported by two methods; comparing inter with intraspecific average nucleotide pairwise identities and via phylogenetic analyses, using reference sequences from GenBank. The mean, between species pairwise nucleotide identity was significantly lower for the Apicomplexa at 71% ± 16.4% (mean ± standard deviation) than the within species means when compared to relevant reference sequences e.g. *H*. *canis* 98.8% ± 0.4% and *B*. *vogeli* 99.0 ± 0.5%. This substantial difference in nucleotide pairwise identity between and within species, at the 18S rRNA region targeted by our primers, highlights how informative this region is for allowing species level classification. Accuracy of classification by our methodology was further supported by phylogenetic analyses. When classified ASVs were aligned with corroborated species reference sequences, they consistently clustered with references of the same taxonomic identity, highlighting an accurate classification (Supplementary File 1; Figs S1 to S4). Overall, this demonstrates the classifier’s ability to recognise high nucleotide homology to reference sequences and accurately assign a species classification to a particular ASV, a tenet that is of particular importance given that such classifiers have been seldom tested on protozoan microbiomes.

During the bioinformatic analysis of the raw NGS data as many as 94.74% of kinetoplastid and 83% of apicomplexan total reads were filtered out at the quality control, denoising, dereplication, pair-joining and chimera removal stage. The large number of lost reads may, in part, be due to the high abundance of host DNA relative to a low quantity of haemoparasite template DNA likely contributing to some off-target deep sequencing, despite the specificity of our designed primers^55,56^. This may have also generated some of the ASVs that were taxonomically identified to groups outside of the primer’s targets, including hits assigned down to the level of Animalia. Nonetheless, even with the raw reads lost through bioinformatic filtering and occasional off-target amplification the NGS diagnostic tool still proved itself more sensitive than endpoint PCR methods.

Financial considerations still limit the use of NGS based methodologies to some degree, however, as costs of deep sequencing reduce, so too does the difference between these methods and cPCR techniques, particularly for studies exploring areas with high levels of infection that necessitate more Sanger sequencing and therefore accrue a higher cost.

Overall, our novel NGS diagnostic tool for the characterisation of the canine haemoparasite microbiome has been demonstrated to be more sensitive and better capable at detecting novel, rare and mixed infections than conventional diagnostic methods. This assay shows much promise in its utility for epidemiological surveys of protozoan haemoparasites, particularly in contexts where parasite diversity and co-infection prevalence is high, such as in developing countries in the tropics. Furthermore, our diagnostic test is not limited to use with canine samples alone and may demonstrate functionality when utilised for the analysis of the haemoparasite microbiome in other animals of veterinary or ecological importance, as well as in humans.

## Methods

### Ethical approval

Ethical approval was obtained from the Animal Ethics Committee of Kasetsart University, Bangkok, Thailand with work conducted under Ethics Permit: OACKU-00758. All experiments were performed in accordance with relevant guidelines and regulations as defined by the University of Melbourne and Kasetsart University, Bangkok.

### Sampling and DNA extraction

This study was part of a larger umbrella project investigating canine and feline VBDs across Bangkok conducted by Kasetsart University, Faculty of Veterinary Medicine. A subset of 100 samples from a total of 1100 collected were used to conduct our NGS diagnostic comparison. Canine blood samples were collected from 35 Buddhist temple communities, after acquisition of informed consent from monks and caregivers. Sampling was done through cephalic or jugular puncture by a qualified veterinarian, collected into EDTA tubes and stored at −20 °C until ready for use. DNA extraction was done using the E.Z.N.A.® Blood DNA Mini Kit (Omega Biotek Inc.) from a starting quantity of 250 µl whole blood according to the manufacturer’s instructions, apart from a reduced final elution volume of 100 µl.

### Apicomplexa and Kinetoplastida 18S rRNA metabarcoding

Sequence alignments for primer design were conducted using Geneious version 11.1.5 (Biomatters Ltd.), whilst Primer3 version 0.4.0 was used to assist in selection of primer sequences. Primer3 parameters were set to amplify regions of approximately 100–200 bps so that they could be successfully amplified by paired-end Illumina sequencing. A large breadth of blood-borne apicomplexan and kinetoplastid genera were chosen for primer design so that our primers had the potential to amplify from as large a range of putative pathogenic organisms as possible. This was particularly important given the exploratory nature of this study and dearth of research into canine haemoparasites in SE Asia. The complete list of sequences used in our alignments are observable in Table 3.

**Table 3:** Parasite and host 18S rRNA sequences used in primer design.

Apicomplexa Primer Design	NCBI Accession Number	Kinetoplastida Primer Design	NCBI Accession Number
Species		Species	
*Babesia gibsoni*	FJ769388	*Leishmania amazonensis*	GQ332354
*Babesia gibsoni*	KC461261	*Leishmania amazonensis*	JX030052
*Babesia vogeli*	KT333456	*Leishmania donovani*	GQ332356
*Babesia sp*. *EU1*	AY046575.1	*Leishmania chagasi*	GQ332357
*Babesia microti*	AB241631.1	*Leishmania infantum*	GQ332359
*Hepatozoon americanum*	AF176836	*Leishmania mexicana*	GQ332360
*Hepatozoon canis*	AY150067	*Leishmania major*	GQ332361
*Hepatozoon sipedon*	JN181157	*Leishmania tropica*	GQ332363
*Hepatozoon felis*	KM435071	*Leishmania aethiopica*	GQ920678
*Theileria velifera*	AF097993	*Trypanosoma cruzi*	CP015675
*Theileria ovis*	AY260172	*Trypanosoma simiae*	AJ404608
*Theileria buffeli*	DQ104611	*Trypanosoma rotatorium*	AJ009161
*Theileria annulata*	EU083801	*Trypanosoma avium*	AF416559
*Theileria sergenti*	EU083802	*Trypanosoma ranarum*	AF119810
*Theileria sinensis*	KF559355	*Trypanosoma neveulemairei*	AF119809
*Plasmodium berghei*	AJ243513	*Trypanosoma mega*	AF119808
*Plasmodium cathemerium*	AY625607	*Trypanosoma chattoni*	AF119807
*Plasmodium ovale*	KF018656	*Trypanosoma fallis*	AF119806
*Plasmodium fragile*	XR001111607	*Trypanosoma evansi*	AY904050.1
*Plasmodium vinckei*	XR552294	*Trypanosoma wauwau*	KT030835
*Toxoplasma gondii*	L24381.1	*Trypanosoma brucei*	XR002989632.1
Canis lupus familiaris*	AAEX03025866	Canis lupus familiaris*	AAEX03025866

Asterisks denotes sequences used to check for primer to host cross-reactivity potential.

The designed primers for Apicomplexa were ApicomplexF: (5′-CRAGGAAGTTTRAGGCAATAACAG-3′) and ApicomplexR: (5′-CTAGGCATTCCTCGTTHAHGATT-3′) which amplify an approximately 130 bp region towards the end of the 18S rRNA gene. The designed Kinetoplastida primers were KinetoF: (5′- CAAACGATGACACCCATGAA-3′) and KinetoR: (5′-CCCCCTGAGACTGTAACCTC-3′) which amplify an approximately 170 bp region in the middle of the 18S rRNA gene. Confirmed positive controls for *B*. *vogeli*, *B*. *gibsoni* and *H*. *canis* were used to optimise and find optimal reaction conditions for the Apicomplexa specific primers. DNA Positive controls for *L*. *infantum* and *T*. *theileri* clade from an Indonesian hill rat, *Bunomys penitus*, were used to find ideal PCR conditions for the Kinetoplastida primers (Table 4). All PCRs were prepared in a PCR hood under aseptic conditions following UV sterilisation. Optimal reaction mixtures for amplification were found to be 20 µl comprising 10 µl of OneTaq® 2X Master Mix with Standard Buffer (New England Biolabs), 0.5 μM of both forward and reverse primers, 1 µl of template DNA and 7 µl of Ambion Nuclease-Free Water (Life Technologies). All PCRs were run with no-template negative controls to check for cross-contamination. Primers were also tested for cross-reactivity against a range of different blood-infecting bacteria, protozoa and metazoa from outside of their target group (Table 4), from which they did not amplify. Optimal thermocycling conditions for the Apicomplexa primers were found to be an initial denaturation of 94 °C for 5 min, followed by 35 cycles of 94 °C for 30 s, 52 °C for 30 s and 72 °C for 30 s with a final elongation at 72 °C for 5 min. Thermocycling conditions for Kinetoplastida primers were initial denaturation of 94 °C for 5 min, followed by 35 cycles of 94 °C for 30 s, 56 °C for 30 s and 72 °C for 30 s with a final elongation at 72 °C for 5 min. During PCR optimisation experiments amplicons were run and visualised on a 1.5% agarose gel using a ChemiDoc™ System (Bio-Rad).

**Table 4:** VBD species positive controls used to test designed primer specificity.

Primer Pair	Species Positive Control	PCR Result	Primer Pair	Species Positive Control	PCR Result
Apicomplexa	*Babesia gibsoni*	**+**	Kinetoplastida	*Leishmania infantum*	**+**
	*Babesia vogeli*	**+**		*Trypanosoma theileri* type 1	**+**
	*Hepatozoon canis*	**+**		*Trypanosoma theileri* type 2	**+**
	*Toxoplasma gondii*	**+**		Babesia gibsoni*	−
	Leishmania infantum*	−		Babesia vogeli*	−
	*Trypanosoma theileri* type 1*	−		Dirofilaria immitis*	−
	Dirofilaria immitis*	−		Rickettsia typhi*	—
	Rickettsia typhi*	−		Anaplasma platys*	−
	Rickettsia felis*	−		Coxiella burnetti*	−
	Anaplasma platys*	−		Mycoplasma haemocanis*	−
	Coxiella burnetti*	−		*Bartonella* spp.*	−
	Mycoplasma haemocanis*	−			

Asterisks denote a VBD outside of the primer’s target group and therefore a test for cross-reactivity.

Deep sequencing of 18S rRNA amplicon metabarcodes was carried out according to Aubrey *et al*.^57^. Briefly, the aforementioned first-step PCR was completed with the addition of overhang sequences at the 5′ end of the Apicomplexa and Kinetoplastida primers. The overhang sequence added to the 5′ end of the forward primer was 5′-GTGACCTATGAACTCAGGAGTC-3′ and to the 5′ end of the reverse primer was 5′-CTGAGACTTGCACATCGCAGC-3′. PCR product was then cleaned using 1X Ampure Beads (Beckman Coulter). A second PCR step was then carried out introducing 8-base forward and reverse indexing sequences, permitting multiplexing of amplicons onto a single run. Sixteen forward indexes and 26 reverse indexes were used allowing multiplexing of 104 Apicomplexa amplicons and 104 Kinetoplastida amplicons. For each target group 100 canine blood DNA samples were run alongside two no-template negative controls and two DNA positive controls that consisted of a previously sequenced unique *B*. *gibsoni* strain for the Apicomplexa PCR and two unique *T*. *theileri* strains for the Kinetoplastida PCR. These unique strains allowed for identification of the appearance of positive control sequences in samples other than controls during NGS data analysis. Thermocycling conditions for this second PCR were an initial denaturation of 95 °C for 2 min, followed by 24 cycles of 95 °C for 15 s, 60 °C for 15 s and 72 °C for 30 s with a final elongation at 72 °C for 7 min. Amplicon size distribution was analysed using an Agilent 2200 Tapestation (Agilent), pooled and then purified using 0.7X Ampure Beads to exclude primer-dimer products^57^. The purified amplicon pool was then diluted using a Qubit 2.0 Fluorometer (Life Technologies) and run on an Illumina MiSeq (Illumina) using 300-cycle v2 chemistry (2 × 150 bp paired-end reads) at the Walter & Eliza Hall Institute Proteomics Facility.

### Bioinformatics

Raw data was demultiplexed using in-house software at the Walter & Eliza Hall Institute. All subsequent bioinformatic analysis was conducted in the QIIME 2 (version 2018.8) environment^58–61^. Primer, adapter and index sequences were trimmed from raw reads using cutadapt^62^ and then imported into the QIIME 2 environment and inspected for quality. DADA2 was then used to remove low quality reads, denoise, dereplicate, filter chimeras and merge forward and reverse reads^63^. Prior observation of read quality plots informed the selection of truncation parameter values when executing the DADA2 program. DADA2 was used to generate Amplicon Sequence Variants (ASVs) instead of Operational Taxonomic Units (OTUs), that provide finer scale resolution of reads^64^. ASVs were then taxonomically assigned using the scikit-learn classifier^65^ against the SILVA version 132 reference database, downloaded from docs.qiime2.org. ASVs were also taxonomically identified using the BLASTn program in GenBank (NCBI) to corroborate scikit-learn assignment and in some cases identify to a lower taxonomic level. Sequences that were unassigned or only assigned to kingdom and phylum or bacterially assigned were excluded from the final dataset. Sequencing depth was validated by generation of alpha rarefaction plots, using MAFFT^66^ and FastTree 2^67^ plugins, to ensure that ASV diversity plateaued and thus a sufficient sequencing depth had been achieved. All NGS data produced in the present study is available from the BioProject database (https://www.ncbi.nlm.nih.gov/bioproject), with BioProjectID: PRJNA528154 and SRA data accession numbers SRR8872668 to SRR8872867.

### Conventional PCR and Sanger sequencing

To compare the sensitivity of our NGS method with traditional molecular techniques all 100 samples were tested for *Babesia* spp. and *H*. *canis* by specific endpoint cPCR screens from the literature (Table 2).

To confirm VBD identification in blood DNA samples by NGS, a subset of samples from different taxon were corroborated by Sanger sequencing. This subset of PCR amplicons was purified using the ExoSAP-IT™ PCR Product Cleanup Reagent kit (Thermo Fisher Scientific) according to the manufacturer’s protocol. Cleaned amplicons were sent to Macrogen (Seoul, South Korea) for Sanger sequencing.

### Statistical analysis

Analysis of results was conducted in Excel 2016 version 1803 (Microsoft), whilst Kappa statistics to compare concordance of NGS vs cPCR results were calculated in SPSS Statistics 24 (IBM).

### Phylogenetic analysis of NGS sequence data

To assess the accuracy of the scikit-learn classifier’s taxonomic assignment, classified ASVs were incorporated into phylogenetic trees to ensure the classification clustered with relevant reference sequences. 18S rRNA sequences were taken from GenBank, aligned with our ASVs in Mega X^68^ and the appropriate primer targeted region extracted. Phylogenetic analyses was conducted using the Bayesian inference (BI) and Monte Carlo Markov Chain (MCMC) method in MrBayes version 3.2.3^69^. The necessary likelihood parameters required for BI analysis were obtained using the Akaike Information Criteria (AIC) test in jModelTest 2 version 2.1.10^70^. To calculate BI posterior probability values, four simultaneous tree-building chains running 2,000,000 iterations were conducted with trees saved every hundred iterations. A 50% majority rule consensus tree for each analysis was constructed based on the final 75% of trees generated by BI. Trees were viewed in FigTree version 1.4.4 (http://tree.bio.ed.ac.uk/software/figtree/).

## Supplementary information

Supplementary File 1

## Data Availability

All NGS data produced in the present study is available from the BioProject database (https://www.ncbi.nlm.nih.gov/bioproject), with BioProjectID: PRJNA528154 and SRA data accession numbers SRR8872668 to SRR8872867.
