# Supplementary material for: A novel metabarcoding diagnostic tool to explore protozoan haemoparasite diversity in mammals: a proof-of-concept study using canines from the tropics
Source: Sci Rep. 2019 Sep 2;9:12644. doi: 10.1038/s41598-019-49118-9 (PMC6718641; doi:10.1038/s41598-019-49118-9)
Supplement: Supplementary file 1 [file 41598_2019_49118_MOESM1_ESM.pdf]

TITLE: A novel metabarcoding diagnostic tool to explore protozoan haemoparasite diversity in mammals: a proof-of-concept study using canines from the tropics

AUTHORS: Lucas G Huggins*[1], Anson V Koehler[1], Dinh Ng-Nguyen[2], Stephen Wilcox[3], Bettina Schunack[4], Tawin Inpankaew[5], Rebecca J Traub[1]

AFFILIATION:

[1]Faculty of Veterinary and Agricultural Sciences, University of Melbourne, Parkville, VIC 3052, Australia

[2]Faculty of Animal Sciences and Veterinary Medicine, Tay Nguyen University, Dak Lak 630000, Vietnam

[3]Walter and Eliza Hall Institute of Medical Research, Parkville, VIC 3052, Australia

[4]Bayer Animal Health GmbH, Leverkusen, Germany

[5]Faculty of Veterinary Medicine, Kasetsart University, Bangkok 10900, Thailand

*Correspondence: lghuggins@student.unimelb.edu.au

Supplementary Figure S1

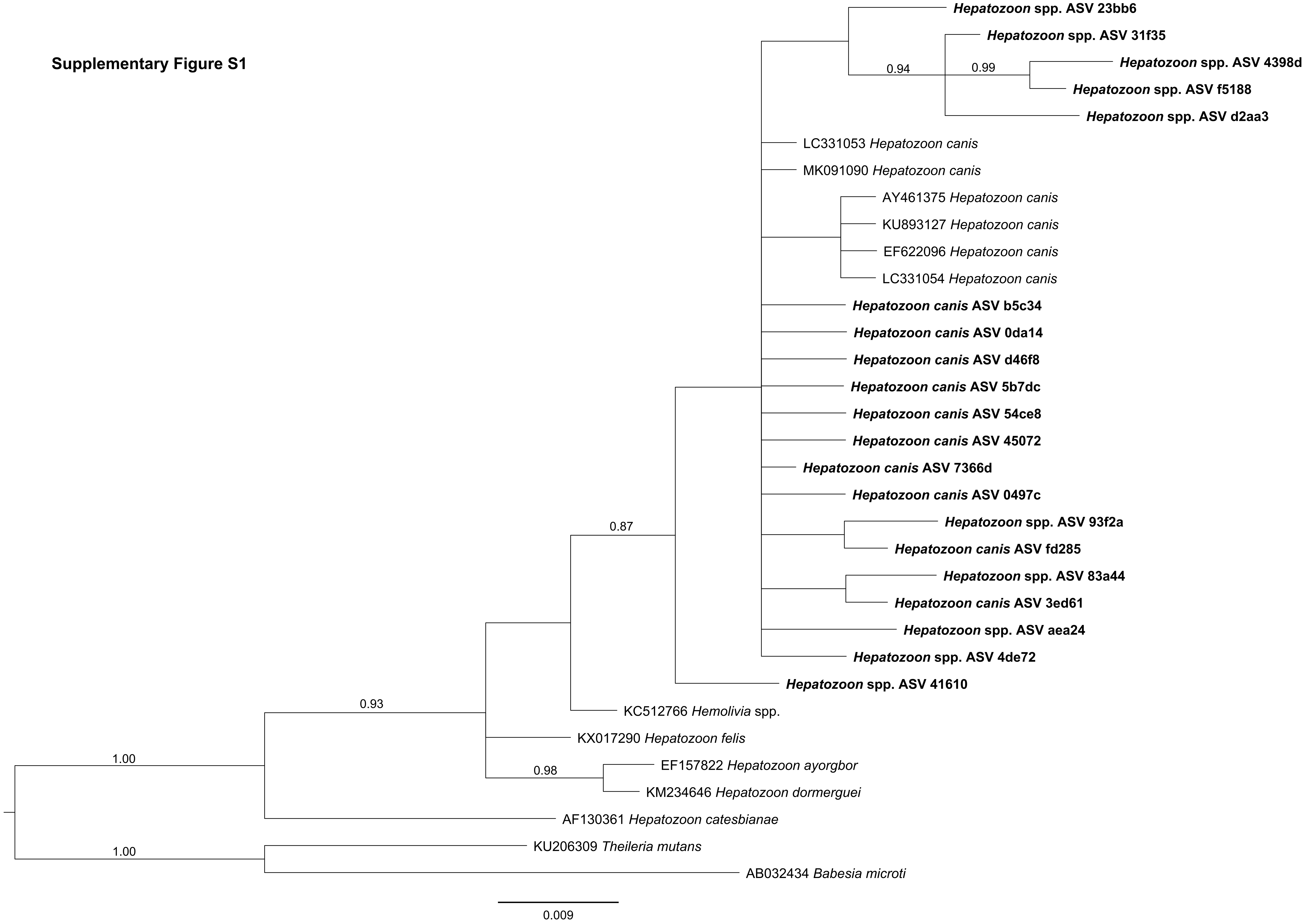

Supplementary Figure S2

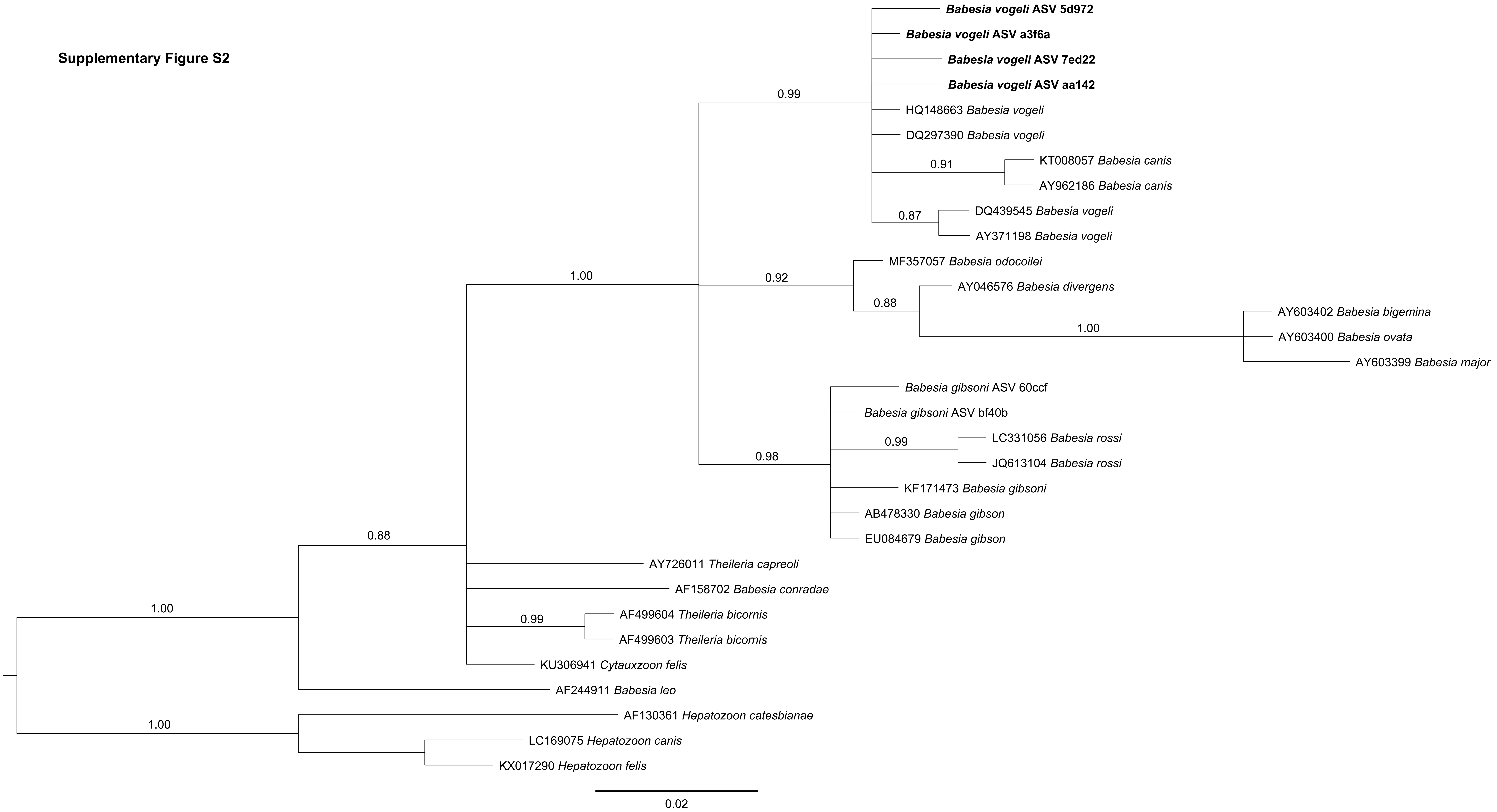

Supplementary Figure S3

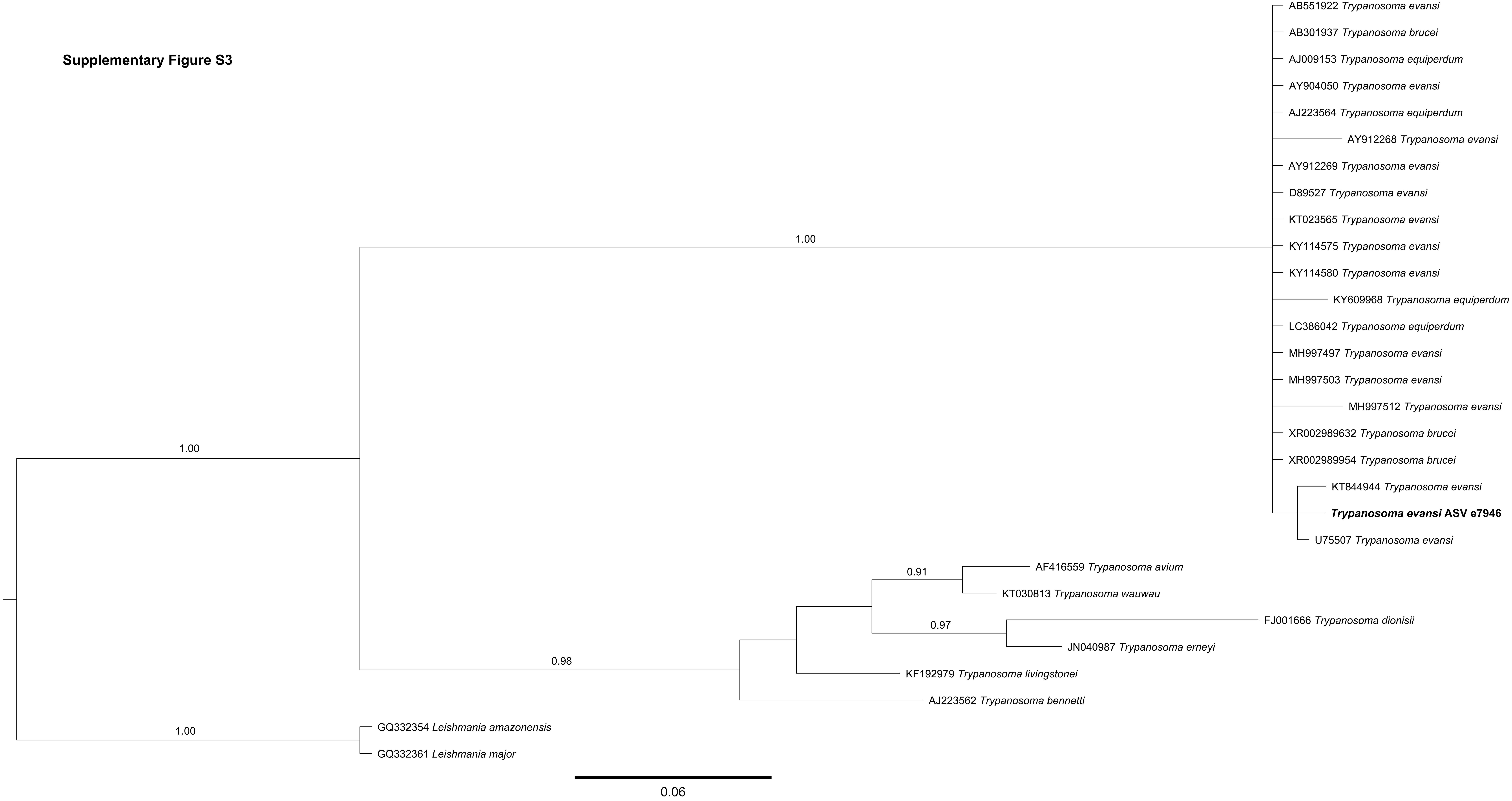

Supplementary Figure S4

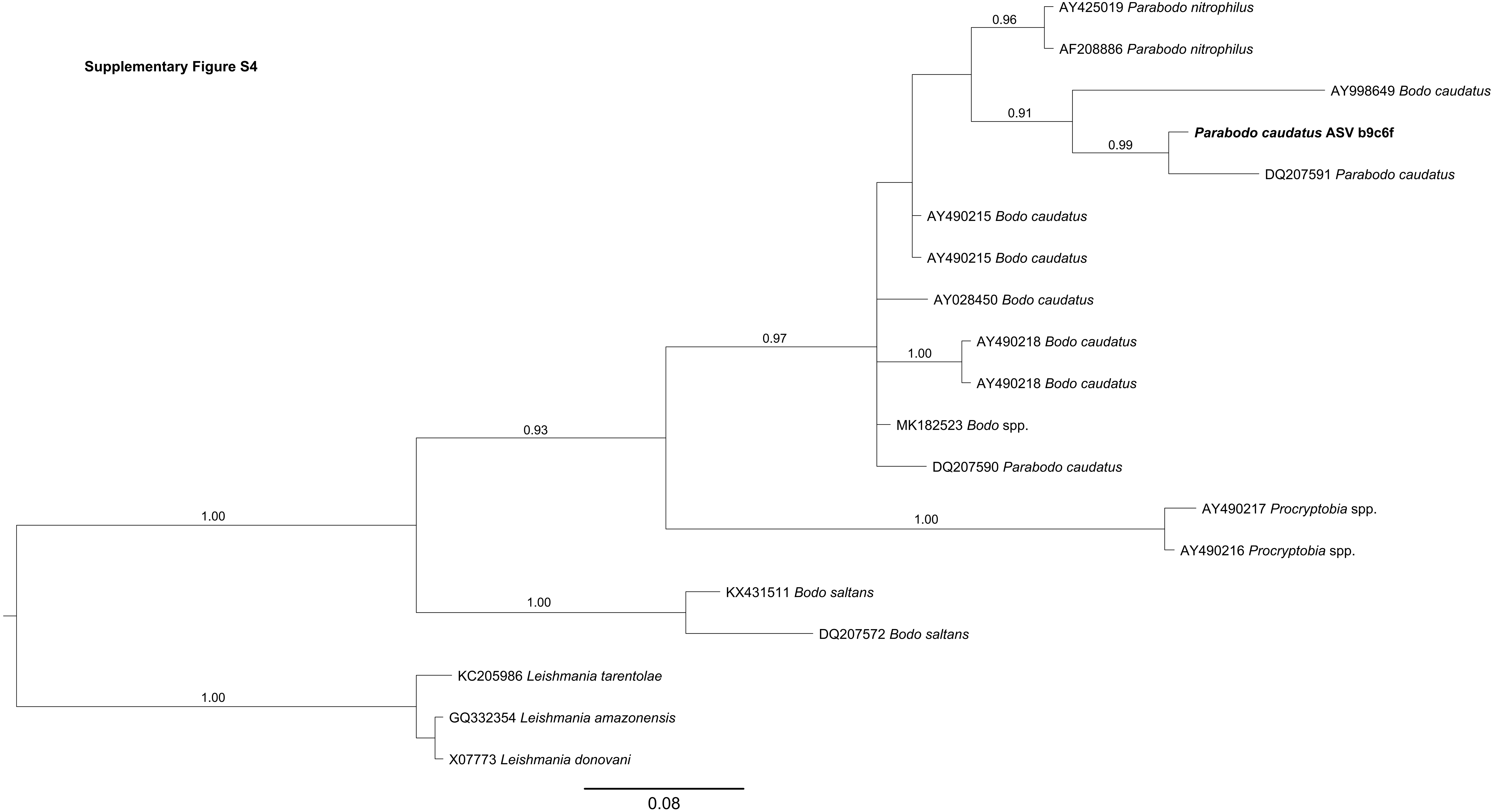

Supplementary file 1: Figure S1. *Hepatozoon* spp. phylogenetic analysis by Bayesian inference (BI) of a 130 bp region of the 18S ribosomal RNA (rRNA) gene targeted by our apicomplexan primers. ASVs assigned as *Hepatozoon* spp. or *Hepatozoon canis* generated from our NGS data are in bold and identified by a 5-character string code. Other sequences are references taken from GenBank, including a variety of *H. canis* sequences from different geographical regions, four closely related *Hepatozoon* species and two piroplasmid species as an outgroup. Posterior probabilities (> 0.85) are indicated on branches. The scale-bar represents the number of substitutions per site. Our *H. canis* classified ASVs cluster within *H. canis* reference sequences. **Figure S2. *Babesia* spp. phylogenetic analysis by Bayesian inference (BI) of a 130 bp region of the 18S ribosomal RNA (rRNA) gene targeted by our apicomplexan primers.** ASVs assigned as *Babesia vogeli* or *Babesia gibsoni* generated from our NGS data are in bold and identified by a 5-character string code. Other sequences are references taken from GenBank, including *B. vogeli* and *B. gibsoni* sequences from different geographical regions. Three *Hepatozoon* species are used as an outgroup, whilst *Theileria* spp. and *Cytauxzoon* spp. are used for comparison. Posterior probabilities (> 0.85) are indicated on branches. The scale-bar represents the number of substitutions per site. Our *B. vogeli* and *B. gibsoni* classified ASVs both cluster next to *B. vogeli* and *B. gibsoni* reference sequences, whilst closely related *Babesia canis* and *Babesia rossi* show a significant number of nucleotide substitutions from their relevant clusters. **Figure S3. *Trypanosoma evansi* phylogenetic analysis by Bayesian inference (BI) of a 170 bp region of the 18S ribosomal RNA (rRNA) gene targeted by our Kinetoplastidae primers.** The ASV assigned as *T. evansi* from our NGS data is in bold and identified by a 5-character string code, other sequences are references taken from GenBank, including *T. evansi* and the closely related *Trypanosoma equiperdum* and *Trypanosoma brucei* from different geographical regions. Two *Leishmania* species are used as an outgroup, whilst a range of more distantly related *Trypanosoma* are used for comparison. Posterior probabilities (> 0.85) are indicated on branches. The scale-bar represents the number of substitutions per site. Our *T. evansi* classified ASV, clusters next to *T. evansi*, *T. equiperdum* and *T. brucei* reference sequences, the latter two are known to be indistinguishable at the 18S rRNA gene, however *T. equiperdum* solely infects horses, whilst *T. brucei* is restricted to Africa, meaning only *T. evansi* can be the responsible pathogen [33,34]. **Figure S4. *Parabodo caudatus* phylogenetic analysis by Bayesian inference (BI) of a 170 bp region of the 18S ribosomal RNA (rRNA) gene targeted by our Kinetoplastidae primers.** The ASV assigned as *P. caudatus* from our NGS data is in bold and identified by a 5-character string code, other sequences are references taken from GenBank, including *P. caudatus* (formerly classified as *Bodo caudatus*) and the closely related *Parabodo nitrophilus* from different geographical regions [36,48]. Three *Leishmania* species are used as an outgroup. Posterior probabilities (> 0.85) are indicated on branches. The scale-bar represents the number of substitutions per site. Our *P. caudatus* classified ASV, clusters next to *P. caudatus* reference sequences with strong posterior probability support.
